# Angina Pectoris

**Published:** 1915-01

**Authors:** Delancey Rochester


### BUFFALO MEDICAL JOURNAL

Yearly Volume 70. JANUARY, 1915. No. 6

### ORIGINAL ARTICLES

The right is reserved to decline papers not dealing with practical medical and surgical subjects, and such as might offend or fail to interest readers. Contributors are solely responsible for opinions, methods of expression and revision of proof.

### Angina Pectoris

By DELANCEY ROCHESTER, M. D.,

(Read before Buffalo Academy of Medicine, 1914)

In speaking to you on Angina Pectoris, I shall be somewhat elementary in my introductory remarks in order to lead up to the correct recognition of the condition and the prognosis and rational treatment of it.

It is, of course, a syndrome, which may accompany many different morbid conditions and it is my purpose to discuss with you the most probable cause of the attacks, that is, the morbid state of the heart, which is always present with an attack of angina pectoris.

Angina pectoris has been defined as an attack of pain in the chest, extending down the arm and forearm, particularly of the left side, accompanied by a constriction of the chest producing a sense of suffocation and also, in most cases, accompanied by a sense of impending death, associated with disease of the heart. The morbid conditions, which are most commonly present are (1) aortic aneurism, (2) aortic valvular disease, especially stenosis, (3) arteriosclerosis with atheroma of the coronary arteris, (4) degeneration of the myocardium, high arterial tension, generally accompanying one or more of the previously mentioned conditions.

In approaching this subject from the standpoint of morbid physiology, we have to consider more than the morbid anatomy for that is constantly present, whereas the attacks come on spasmodically—with nothing regular about their occurence. What then are the conditions which give rise to the attacks? Emotional excitement, physical exertion and exposure to cold. Anyone of these, or any combination of them can readily bring about the attack. If we analyze these conditions we find that the quality which they have in common is that, through raising peripheral tension, they throw extra work on the left ventricle of the heart which is in a morbidly weak state.

Now I must ask you to review with me the physiology of the heart as revealed by the studies of the circulation in the last five years. The function of the muscular fibres of the heart have been shown to be five, viz., stimulus production, excitability, or power to receive a stimulus, conductivity or power to transmit the stimulus to other fibres, contractility or power to contract under stimulus, and tonicity or power to retain a certain amount of contractile tone even when active contraction has ceased.

In the majority of cases of heart disease with which angina pectoris is associated the first three of these muscle fibre functions, stimulus production, excitability, conductivity are unimpaired. There remain the functions of tonicity and contractility. When these two functions are impaired there results dilatation of heart. In by far the majority of cases with which angina occurs there is present dilatation of left ventricle. There are, however, some cases in which dilatation cannot be demonstrated, so that, while in most cases tonicity is impaired, the fact that angina can occur without its impairment throws us back on the last function—contractility as the one with the interference with which angina is always present. Then you will say this throws us back on the old theory of heart spasm as the morbid condition present during angina, but as a matter of fact, muscle spasm, such as we see in the case of the hard contracting uterus is impossible in the heart for that would cause complete cessation of cardiac activity and, of course, death. We can believe, however, and are forced so to do that pain can be induced by the attempt on the part of a hollow viscus to contract forcibly against resistance. We certainly have evidence of this in the pain produced when an intestine contracts in the case of obstruction of the bowel. That this may explain the production of pain is evident, but why the peculiar distribution of the pain?

Here we have to recur to developmental anatomy to explain the distribution of the pain and to morbid physiology to explain the origin and cause of the pain and chest constriction and sense of suffocation.

To refer once more to the intestines. In several instances where incision has been made into the abdomen under local anaesthesia, so that pain is not produced by the incision and the bowel has been put on the outside of the abdominal wall. marked contraction of the bowel in peristalsis has occurred and the patient has complained of pain. When asked to locate the pain, invariably the location of the pain has been in some part of the abdominal wall, usually in the neighborhood of the umbilicus and not in the contracting intestine. This phenomenon is known as a viscero-sensory reflex and has been shown to be due to the carrying in of the stimulus of the contraction of the intestine by an afferent nerve to the spinal cord and hence to the brain and this stimulation being sent out thru a sensory efferent nerve to the region to which its filaments are distributed and its expression as pain.

We have also the well recognized muscular spasm of the abdominal wall in case of underlying visceral disease, as cholecystitis, appendicitis, etc. This phenomenon is known as a viscero-motor reflex and is explained in the same way except that the efferent stimulus is borne out on a motor nerve causing muscular contraction of the overlying wall instead of on a sensory nerve producing pain.

That muscle spasm, a viscero-motor reflex, has, as its purpose, protection, is plain. Hilton, in his excellent monograph on "Rest and Pain" has pointed out that the pain of viscero-sensory reflex origin also has, as its purpose, protection in that it demands cessation of any action which causes it.

The why and wherefore of surface pain and of muscular spasm of overlying body wall are, I think, thus properly explained.

The reason why the pain in angina pectoris has its peculiar distribution can be explained if we consider the origin of the cerebro spinal and autonomic innervation of the heart and of the parts where the pain is felt. Both have their innervation originally from the same sources, so when a stimulus from an overworked organ is sent in on an afferent nerve the corresponding stimulus is sent out as pain or muscular contraction to the parts supplied. The nerve supply to all these parts comes originally from the last cervical and the first, second and third dorsal spinal nerves and from certain of the cerebral nerves. The sensory filaments from these spinal nerves are distributed to the fore-arm, arm and second and third intercostal spaces, where the pain is felt. The motor filaments concerned are those distributed to the second, third and fourth intercostal muscles, causing them to contract and produce the sensation of constriction and suffocation.

So far as I know the only other condition, which can produce the peculiar distribution of pain referred to, is herpes zoster of those nerves. This eventually shows itself in the eruption. So I believe that we can put down at least 95 per cent. of cases of pain in these regions as of cardiac origin. The attack does not always follow immediately the exciting cause but may appear several hours later even after the exciting cause has ceased to be active. This has been so well explained by MacKenzie as being due to a summation of stimuli, that I cannot do better than to quote:

"The fundamental functions of the heart muscle correspond to those of other involuntary muscles that form the walls of hollow organs; these functions being modified to suit its special work. Like the other viscera, the heart is insensitive when stimulated in a manner that provokes pain when applied to the tissues of the external body-wall. I may point out that a prolonged strong contraction of a hollow organ can produce pain, and that this undoubtedly is the cause of the severe pain associated with renal calculus, gall-stones, spasm of the bowel, and uterine contractions. Can the heart give rise to pain in a similar manner? On account of the modification of its functions, the heart cannot pass into a prolonged state of contraction. Immediately it contracts, the function of contractility is abolished and the muscle passes at once into a state of relaxation, and for this reason the pain cannot be produced by a 'spasm of the heart.' But I suggest that the heart muscle may produce pain when it is confronted with work greater than what it can readily overcome—a condition which produces strong peristalsis and pain in other hollow viscera. But the pain in the heart arises by a slightly different mechanism. A skeletal muscle will contract in obedience to stimulation of a sensory nerve going to the spinal centre of its nerve, if a stimulus of sufficient strength be applied. If the stimulus is too weak no contraction follows, but if this weak stimulus be frequently and rapidly repeated, then the muscle contracts in accordance with the law of the summation of stimuli. I suggest that the heart muscle induces pain on the principle of summation of the stimuli. If we minutely study our cases we shall find that the pain rarely arises at the first exposure of the heart to the effort that induces pain; sometimes effort has been undertaken a few minutes before pain comes on, and in certain cases it may not come on for hours after the casual exertion has ceased. From such observation we can infer that the heart muscle was exhausted by the exertion, and so great was the exhaustion of the reserve force that the heart was unable to regain its reserve with cessation of effort, so that the exhaustion persisted till it culminated in an attack of angina pectoris. The conditions predisposing to attack are then any condition which has lasted long enough to produce a weakening of the left ventricle wall and causing this wall to be put upon severe strain under exciting cause. All physicians of sufficient experience have noted that in some cases, subject to angina pectoris, in which a mitral insufficiency develops, the attacks cease when by this means the great strain is taken off the left ventricle by the yielding of the valve, through dilatation of the auriculo-ventricular opening. The conditions producing this weakness of the left ventricular wall are, in my opinion, always some form of arteriosclerosis, affecting either the coronaries and so interfering with the proper nutrition of the myocardium, or occurring in the aorta either at the valve producing stenosis or beyond causing inelasticity of aortic wall or further along in some of the peripheral arteries, especially those of the kidney—raising the tension permanently against which the heart has to work."

Of the symptoms of angina pectoris, the one which is always present is pain. The peculiar distribution of this pain and the mode of its production have been thoroughly discussed.

In at least 95 per cent of cases, which show this distribution of pain, the origin of the pain is in the heart.

The pain varies greatly in its intensity and in its duration. If the proposition is true that the pain is produced by the attempt at contraction against a resistance, which for the moment is too great, we can readily understand that it may occur in cases in which the myocardium is in pretty fair state but the peripheral resistance is too great for it. I recall one case in which there was no evidence of real disease of the heart but there was a high peripheral pressure from a marked pyelonephritis and the patient had been under a severe psychic strain, when he was put to it by a disturbing letter read at the end of an exhausting days work.

He was seized with the typical pain together with the sense of chest constriction which lasted about 15 minutes, leaving him depressed mentally and physically. About one hour later under the physical strain of mounting rather a long flight of stairs the symptoms returned, lasting about thirty minutes. He has not had a return of the attack though this was 5 or 6 years ago.

Some may say that this was pseudo-angina. The term pseudo-angina, in my opinion, had better be dropped. Either the pain is of cardiac origin or it is not. If of cardiac origin, due to this attempt at contraction against resistance, it is angina, even though it may not recur and disease of heart cannot be demonstrated by physical examination. The pain may be of this mild evanescent character or may be of any grade of severity up to the point of anguish, which is usually given as the typical angina attack. The sense of constriction of the chest and suffocation is associated with a great many of the angina attacks, the sense of impending death is not as common, and, in my experience has never been present except in association with both of the other symptoms.

I recall the case of a man who had been the subject of angina attacks for several years, generally they yielded to nitrite amyl inhalation and the administration of nitro-glycerin.

I was called to see him one afternoon in February. He had been feeling pretty well that day, and on returning from the office had taken a street car to within two blocks of his home and had walked that short distance in the cold, against the wind. He had had to stop three times before he reached his home and after resting there down stairs for a short period had mounted the stairs and when he reached his room was seized. When I arrived, he was sitting on a chair grasping the arms in both hands, his collar was unbuttoned, his face was a bluish pallor, his forehead covered with a cold sweat, his mouth open and he was gasping for breath, with an expression of suffering and anguish upon his countenance which was most distressing to observe. There was the odor of amyl nitrite about and he had taken in divided doses 1/25 grain of nitro glycerin in one hour without relief.

In this case, as in all severe attacks, the gripping of the chest as though the breast bone would break adds tremendously to the suffering; especially as it usually develops after the pain and both increase in severity together.

The fear of impending death is in all probability of psychic origin and is brought about by the presence of pain and anguish referred to the region with which the continuance of existence has always been associated. Patients do occasionally die during an attack of angina pectoris, but death under such circumstances, is not common when we think of the great number of attacks of angina which occur without such termination.

Having thus briefly reviewed with you the mode of production of attacks of angina pectoris, I think that we are justified in the statement that in at least 90 per cent. of cases in which angina pectoris occurs, arteriosclerosis is present in some form or another, and in one or more places that it is quite advanced, that in the vast majority of cases it is present in the aorta, the aortic valves and the coronary arteries; that in the 10 per cent. of cases in which arteriosclerosis cannot be demonstrated there is always some morbid process present which has produced a certain amount of toxic weakness in the myocardium at the same time raising the peripheral tension, as in the case of the pyelo-nephritis already referred to.

As the etiology, diagnosis, prognosis and treatment are so closely associated, I shall consider them together.

First as to diagnosis: if my premises are correct I think we may make the diagnosis very easily.

The peculiar distribution of the pain when there is not local cause such as growth pressing upon the nerves or injury which might cause it, especially if it has associated with it a gripping of the chest, is sufficient.

When we consider that arterio-sclerosis is so frequent a causative factor of angina pectoris, we are brought up against the fact that all the varied causes of arterio-sclerosis have to be considered in the etiology of this syndrome.

The chief of these are syphilis, hard muscular work and chronic, commonly autogenous, toxaemias.

Therefore, in every case in which we have to consider the prognosis and treatment we must always get at the chief etiological factor.

In those cases in which syphilis can be shown to be at the bottom of the arterial change and heart degeneration (the Wassermann reaction should always be looked for in cases of angina,) the outlook is not so bad, if the degeneration has not progressed too far. I have been surprised at the improvement of such cases under the use of salvarsan and hypodermic administration of mercury.

If syphilis be not present, and it is possible to remove the active cause of the arterio-sclerosis, as for example by changing the occupation and mode of life, good results sometimes come from institution of such treatment.

The prognosis is based upon the chief etiological factor and the ability to modify it, upon the degree to which degeneration has proceeded in the arteries and in the myocardium and upon any accompanying complicating disease.

If we now revert to the physiologic ground of the attack, viz., the attempt of the myocardium to contract against an opposing pressure which is too great, for the strength of the myocardium, the prognosis will be greatly modified by the conditions present. It will take less obstructing pressure to produce an attack in a very weak myocardium than in a stronger one. So we may say that generally the prognosis is worse when angina occurs in cases with relatively low blood pressure, both for the relief of the attack and for the recovery of the patient. The pressure being already low we have to be careful how we use measures that will lower it still further, so that in such cases I generally feel my way with cardiac stimulants such as strychnine and digitalis or caffein in combination with vaso-dilators.

Therefore, it is most important to recognize the morbid condition present before making a prognosis or laying out a plan of treatment in a given case.

I think I cannot do better than to cite several cases to illustrate what I mean by the different plans of treatment that must be adopted in different cases. The treatment divides naturally into two parts, viz., the treatment during the attack and the treatment of the morbid state which has led up to the attack. As for the attack, recognizing that physical over exertion, emotional excitement and exposure to cold are the elements which enter into the production of the attack, our energies should be first devoted to producing the opposite state of affairs. We should put the patient at physical rest, induce composure and apply warmth to the surface of the body, especially to the extremities.

I recall one case of a man of fifty, to whom I was called one evening. The history was as follows: Occupation traveling salesman, married, in his early life had worked hard on a farm for twenty years. Gave up this occupation some ten years previously because after an unusually hard day's work in the hay field, he had lifted a heavy piece of ice into the ice box and had had a collapse from which he did not recover in four months. Since that time if he did any strenuous work or became chilled he would have an attack of pain in his chest. There was denial of any venereal disease and he was not under observation long enough to take the blood for the Wassermann reaction. On this occasion he had taken a long ride in the cold in a cutter and after arriving at the hotel, although he felt a little mean as he expressed it, he ate rather a hearty meal. About one hour after he felt distressed and nauseated and forced himself to vomit.

However, the unloading the stomach did not relieve him, the pain gradually grew worse and I was sent for.

I found that he had had previous attacks similar in nature, but none as severe as the present one. The pain and the gripping in the chest were characteristic. His pulse was 100, rather small, regular. Sys. Blood 165 mmhg., temperature 97° F., his feet and hands were cold. The physical examination revealed a heart moderately hypertrophied with a harsh systolic murmur at the aortic area and a sharp closure of the valve. The first sound of the heart accompanying the murmur was not very strong and was rather short.

I gave him a few whiffs of amyl nitrite, which did not help him, and then I ordered his feet put in hot mustard water and applied heat to his body in the form of bottles filled with hot water. Five minuter after his feet were put into the mustard foot-bath he began to feel relieved and in 20 minutes he was comfortable. His blood pressure having fallen to 150. This case did not remain under my care, so I do not know his further history.

This case illustrates the value of the application of surface heat to the body in causing dilatation of the capillaries in reducing the blood pressure against which his weakened heart was struggling.

The next case is important as illustrating the fact that we have no standard of blood pressure applicable to all cases.

A single woman between sixty and seventy years of age, with a previous medical history of no significance, except that she for years had been a very large feeder and had taken very little exercise, has been under my observation off and on for the last twenty-seven years, about ten years ago she developed a dietetic glycosuria, which disappeared under appropriate feeding and has reappeared but once two years after the first. She has been the subject of interstitial nephritis for the last eight years, and has consequently taken pretty good care of herself, in the line of sweat-baths, diet, etc. Her blood pressure, under which she is most comfortable is 200 mmhg. Six years ago in February, she did considerable walking in the shops down town and riding home in a sleigh became chilled. When she arrived at the house, she climbed quite a long flight of stairs and was seized with an attack of pain and gripping in her chest and faintness, with a very decided fear of impending death.

When I called to see her, she was in collapse, her features pinched, breathing labored, pulse small and frequent, heart slightly dilated, first sound of heart short and weak accompanied by a slight mitral insufficiency. Blood pressure 165 mmhg. She had already taken a large dose of whiskey in hot water and after being put to bed and covered up warm still kept up the pain in her chest and it extended to the forearm. Her breathing was labored and she was slightly cyanotic. Remembering her usual blood pressure, under which she was comfortable, I argued that the pain had come on from the increase of blood pressure due to her exertion and the cold, that the myocardium was not strong enough to overcome the increased peripheral pressure and that we were having an acute dilatation of the heart—that the pain was produced through the efforts at contraction which were proving ineffectual and that if we were going to bring her through we should have to not only administer vaso dilators but also someone of the digitalis group to help the contraction of the myocardium. She was accordingly given a combination of Sodium Nitrite, Tincture Digitalis and Fluid Extract Valerian. Her bowels were emptied by the use of an enema of Epsom Salts and Glycerin. Under this treatment her pain gradually ceased, her color returned to normal, in fact, all her symptoms of a serious nature disappeared. When, at the end of four hours, she was feeling well I took her blood pressure again and found that it had increased and was now 190 mmhg.

Here were two cases in one of which 165 mmhg., was an abnormally high pressure and in the other the same pressure was a dangerously low one.

I have had several cases subject to attacks of angina pectoris with high blood pressure, who during one of the attacks have developed mitral insufficiency with the subsidence of the attack and no recurrence of same but the gradual development of dropsies and eventual death from uncompensated dilatation.

Interesting in this respect are two other cases, in each of which the attack of angina disappeared with the development of mitral insufficiency, but in these cases under appropriate treatment compensation was re-established and the mitral insufficiency disappeared, but the tendency to the recurrence of the attacks of angina pectoris also returned. In one case this was so marked that the patient said that he wished that I had let him die.

I have another case—a man of 65 called me one night to see him on account of severe pain in his chest and a tremendous fear of death.

I found him sitting in a chair with a pale face slightly cyanotic with cold sweat standing out on it. His pulse was 110 irregularly intermittent, his blood pressure was 140 mmhg., and still he was suffering from a typical attack of angina pectoris. His heart was dilated, there was present a systolis mitral murmur and a very rough aortic first sound followed by a sharp aortic closure, a systolic murmur was plainly perceptible in the carotids. Here was another case in which mitral insufficiency and aortic obstruction together reduced the bulk of blood passing into the general circulation and into the carotids so that what was a moderate pressure for a man of his years was too high for his weak myocardium. It would have made matters much worse for him to have given him digitalis or any of its congeners. So I gave him a small hypodermic of morphine with nitro glycerin and repeated both in half hour. The two injections, amounting to gr. 1/6 of morphine and gr. 1/50 of nitro glycerin, did the trick and he was comfortable.

In cases of aortic stenosis in which angina pectoris develops, I have never succeeded in relieving the attack without morphine. I always combine nitro glycerin with it, because in all such cases I think there is the element of arteriole constriction besides the stenosis acting against the contracting ventricle.

By the citation of these cases I do not wish to be understood as not advocating the use of amyl nitrite and nitro glycerin during most attacks. In by far the large majority of cases it is the plan above all others which is successful, what I do wish to impress is the importance of studying each case on its own merit and to treat the case not the symptoms. So much for the treatment of the attacks. The management of the case, so that the attacks do not occur, is in my mind the most important thing. Therefore, in all cases in which angina has occured, whether there is evidence of aortic orifice diseases or not, I strongly urge the investigation of the Wassermann reaction. If it is positive the administration of Salvarsan and a course of Mercury hypodermically alternating with potass iodide is indicated. In this way in many cases the recurrence of the attacks may be prevented. In most cases the careful supervision of the diet, the use of the minimum of meat proteids, sometimes the complete elimination of them, is necessary, and keeping the whole diet down to the minimum compatible with the health of the individual, and the absolute prohibition of alcoholics excepting in medicinal doses at specified times, the full use of elimination by skin and bowels and the rendering the urine bland through the use of alkalies. The exercise of the individual to be carefully supervised.

The regular administration in small doses of iodide and nitrite.

In many cases, especially in women, before the occurrence of the attack, there not infrequently occurs a period of sleeplessness and nervousness, which should be treated with Ammon, Bromide in sufficient dose to produce sleep and quiet the nervousness. I generally administer 10 or 15 grains t.i.d. and one large dose of 30 or 45 grains at night.

Under some such general plan of management of life and careful medical supervision, the patient seeing the physician once a fortnight or so, the attacks are certainly diminished in number and severity and the patient given years of comfort.

---

**Notifiable Diseases: Prevalence During 1913.** Reprint No. 210 Public Health Service Reports. Typhoid shows a mortality rate varying from 3.48 (4 deaths in 115 cases) for Paterson, N. J. to 47.97 (59 deaths in 123 cases) for Kansas City. We doubt very much whether typhoid has so marked a fluctuation in virulence. It is easy to account for an abnormally high death rate by failure to report mild cases; not so easy to account for an abnormally low mortality although one may suggest confusion with para-infections in epidemic form or some defect of routine Widal tests. Most cities show a mortality from 10 to 20%, the former being usually considered a fair average, the latter due to failure to report mild cases. The highest urban (populations over 100,000) incidence was 2.716: 1000 population for Albany, which, it will be remembered, had an accidental contamination of its filter beds; the lowest was 0.379 for Milwaukee but as this city gives a death rate of 29.13, we may suspect that its true incidence was about 1: 1000. Buffalo had an incidence of 0.676, mortality 22.52; Rochester 0.602 and 16.90; Syracuse 0.833 and 15.57 respectively. Really, the ultimate mortality per 1000 population is the only fair test of sanitary conditions and even then, we must allow for differences in imported cases. For instance, in Paris, practically all cases come from the suburbs and in Buffalo, it appears that 50-75% of the cases are imported or due to extramural infection. It is difficult to draw lessons from the incidence and mortality of other diseases.

---

**Iodine and Freezing Treatment of Ringworm.** Foley, London Lancet, washes the part with a strong solution of sodium bicarbonate, then with ether to remove fat, paints with tr. iodi and freezes with ethyl chlorid. He reports cures in all cases within a week.

